# Salvage high intensity focused ultrasound for residual or recurrent cervical cancer after definitive chemoradiotherapy

**DOI:** 10.3389/fimmu.2022.995930

**Published:** 2022-10-17

**Authors:** Qin Zhong, Fei Tang, Tingting Ni, Yanping Chen, Yuncong Liu, Jing Wu, Wen Zhou, Zhiyu Feng, Xiaokai Lu, Shisheng Tan, Yu Zhang

**Affiliations:** ^1^ Department of Medical Oncology, Guizhou Province People’s Hospital, Guiyang, China; ^2^ National Health Commission Key Laboratory of Pulmonary Immune-Related Diseases, Guizhou Province People's Hospital, Guiyang, Guizhou, China

**Keywords:** high-intensity focused ultrasound, cervical cancer, ablation, residual diseases, neoplasm recurrence

## Abstract

**Objective:**

The treatment of residual/recurrent cervical cancer within a previously irradiated area is challenging and generally associated with a poor outcome. Local treatments such as salvage surgery and re-irradiation are usually traumatic and have limited efficacy. High intensity focused ultrasound (HIFU) treatment can directly ablate solid tumors without damaging neighboring healthy tissue. However, the HIFU studies for these patients are limited. Experience gained over the course of 10 years with the use of HIFU for the management of residual/recurrent cervical cancer after chemoradiotherapy is reported herein.

**Methods:**

153 patients with residual/recurrent cervical cancer in a previously irradiated field who received HIFU treatment between 2010 and 2021 were retrospectively analyzed. Adverse effects, survival benefit and factors affecting prognosis were given particular attention.

**Results:**

A total of 36 patients (23.5%) achieved a partial response following HIFU treatment and 107 patients (69.9%) had stable disease. The objective response and disease control rates were 23.5% and 93.5%, respectively. The median progression-free survival (mPFS) and median overall survival (mOS) were 17.0 months and 24.5 months, respectively. Moreover, patients with lesions ≥1.40 cm before HIFU treatment and a shrinkage rate ≥ 30% after treatment had a higher mPFS and mOS, and patients with lesions ≤1.00 cm after HIFU treatment had a higher mPFS (P=<0.05). All the treatment-related adverse events were limited to minor complications, which included skin burns, abdominal pain and vaginal discharge.

**Conclusions:**

HIFU treatment is likely a preferred option for cervical cancer patients with residual disease or recurrence following CRT that can safely improve the local control rate and extend survival.

## Introduction

Cervical cancer is one of the most common gynecological malignancies. There were approximately 570,000 newly diagnosed cases and about 311,000 deaths cases of cervical cancer in women worldwide in 2018 (1). Chemoradiotherapy (CRT) has been recognized as the standard treatment for patients with locally advanced cervical cancer (LACC) (2). However, approximately 30%-65% of patients have residual disease or subsequent recurrence after completing CRT (3–5). Local residual disease and recurrence are important factors that contribute to treatment failure and the poor prognosis for LACC patients (6). The treatment of cervical cancer following residual disease or recurrence represents a challenging problem. Thus, research is required to provide safe and effective treatments for patients with radiation resistant cervical cancer. Furthermore, the administration of radiotherapy is usually chosen cautiously due to the side effects associated with the treatment and the dose limitation for healthy tissue.

Systemic platinum-based dual-drug combination salvage chemotherapy is often considered to be an important treatment option for these patients, such as cisplatin combined with paclitaxel or topotecan. Unfortunately, cervical cancer patients with residual disease or recurrence following CRT often face a poor prognosis following the chemotherapy (7–10). A recent multicenter retrospective study reported the median progression-free survival (mPFS) and median overall survival (mOS) following systemic chemotherapy in patients with persistent cervical cancer after CRT to be 8.4 months and 18.0 months, respectively (11). A number of reasons for the poor efficacy have been proposed, including 1) that the prior radiation therapy limited the drug distribution to the tumor, 2) an intrinsic chemoresistance of residual or recurrent lesions, 3) poor bone marrow and/or kidney function in these patients that reduces their tolerance to chemotherapy (7). Nevertheless, the addition of bevacizumab to the platinum-based doublet chemotherapy, has been reported to reduce the risk of death for these patients by 27%. However, this treatment schedule was associated with a 15% fistula occurrence (12).

A local therapy (as opposed to a systemic therapy) has often been considered to be a better choice for cervical cancer patients with local residual disease or recurrence after CRT. Pelvic exenteration or radical hysterectomy has been offered as an optional treatment, however, the benefit for these patients remains controversial. The published five-year survival rate following surgery for patients with residual disease or recurrence following CRT ranges from 20% to 73% (4, 6, 11, 13–15). Overall, surgery appears to offer a significant improvement for patients, however, surgery after CRT is difficult due to the risk of serious pelvic adhesions, poor tissue recovery, unclear anatomy and excessive local blood supply. Moreover, the frequency of serious complications is high (15-25% chance of adverse events ≥grade 2, including postoperative death), which leads some patients to refuse surgery (6, 11, 16, 17). Ota et al. (18) reported that out of 162 patients with persistent local disease following CRT only 35 (21.6%) opted for a hysterectomy. The majority of patients were considered inoperable, 62.3% of patients were not operated on due to their advanced age, poor medical condition or refusal of surgery, and 16.0% could not receive surgery due to concomitant distant metastasis.

High intensity focused ultrasound (HIFU) is a method for solid tissue ablation using focused ultrasound energy (19). In recent years, HIFU has been increasingly used to treat malignant tumors, including: liver, pancreatic and prostate cancer, especially for tumors that lack other effective treatment methods. A number of studies have confirmed the effectiveness, safety and feasibility of HIFU for the treatment of malignant tumors (20, 21). HIFU is a non-invasive technique that can focus on a specific treatment area with a clear boundary from the non-treatment area, which enables the preservation of tumor adjacent healthy tissues (22). The fact that few side effects are caused by HIFU make the technique a good option for patients who have received pelvic CRT, especially those with severe pelvic adhesions and those unable to tolerate an additional high-intensity systemic treatment. However, there are few reports describing the use of HIFU treatment for cervical cancer (23, 24). Therefore, the study contained herein details the use of HIFU for 153 cervical cancer patients that experienced disease persistence or recurrence following CRT. The experience gained from this treatment schedule over more than 10 years is reported with particular attention to adverse effects, survival benefit and factors affecting prognosis.

## Materials and methods

### Patients characteristics

This retrospective analysis of cervical cancer patients who underwent radiotherapy (included either radiotherapy after radical hysterectomy or definitive radiotherapy with or without chemotherapy) and experienced tumor persistence or recurrence in a previously irradiated field from January 2010 to January 2021 at the Guizhou Provincial People’s Hospital (CN). The inclusion criteria were as follows: (1) an age of ≥18 years; (2) patients with cervical cancer disease persistence/recurrence after CRT that was confirmed using at least two imaging techniques (such as computed tomography (CT), magnetic resonance imaging (MRI) or positron emission tomography (PET) with [^18^F]-fluorodeoxyglucose, with one occurring no more than two weeks prior to the HIFU treatment) and with histological (or cytological) confirmation for those in accessible sites; (3) those with malignant lesions that were localized only within the pelvic cavity; (4) patients who either refused or were unsuitable for salvage surgery and radiotherapy; (5) an Eastern Cooperative Oncology Group (ECOG) performance status of 0-2; (6) sufficient organ function (a neutrophil count ≥1.5 × 10^9^/L; hemoglobin  ≥8 g/dL; platelets ≥75 × 10^9^/L; AST/ALT ≤5 × normal value; creatinine within the normal range or creatinine clearance ≥50  mL/min); (7) patients with lesions for treatment that were large enough to be sufficiently visible using ultrasound; (8) the provision of signed informed consent. The exclusion criteria were: (1) extensive pelvic lesions or lesions beyond the pelvic cavity; (2) an ECOG performance status ≥3; (3) patients with lesions that could not be visualized, or with lesions that were unable to be targeted by the focus range; (4) pronounced scarring along the acoustic path. The clinicopathological data and the treatment details for the aforementioned patients were also collected.

### HIFU therapeutic procedure

HIFU ablation was performed with a high intensity focused tumor therapy system (model HIFU-2001, Shanghai Jiao Tong University’s Xindi Industrial Company, CN) equipped with a real time ultrasound guidance device. The therapeutic-focused ultrasonic working frequency was set to 50 Hz with an output power of 1kW and degassed water as a treatment medium. The effective treatment depth was 10 mm-150 mm, a focal volume of 3 mm × 3 mm × 8 mm and an effect focus of 6 mm × 6 mm × 10 mm. The parameters of HIFU treatment were adjusted for each patient according to the location and depth of the tumor, the tumor tissue density and the sound attenuation rate. The treatment array was formed by point accumulation with fractional treatment for large volumes of lesions.

All patients consumed semi-liquid/liquid food for 2-3 days, fasted for 12 hours and received an enema prior to the HIFU treatment. The patients were positioned prone on the HIFU treatment table with the abdominal wall in contact with degassed water. Their breathing, heart rate, blood pressure and oxygen saturation were monitored throughout treatment. The distance of the target tumor from the skin was measured using B-ultrasound, which was marked on the skin. The B-ultrasound probe was used to move the focus to the deepest part of the tumor and the treatment area was delineated. The treatment area was computationally marked by the displacement of the treatment basin in the form of dots, lines and surfaces, after which the HIFU treatment was automatically completed by the HIFU system under the supervision of 1-2 doctors.

### Evaluation of therapeutic efficacy and survival

Four to eight weeks following the HIFU treatment, the patients received imaging with CT, MRI or PET with [^18^F]-fluorodeoxyglucose, and a follow up appointment was performed every 2-3 months, including a gynecological examination and cytology if necessary. The tumor response was defined by the response evaluation criteria in solid tumors (RECIST) version 1.1 (25, 26). The response for each target lesion was classified as having a complete response (CR), partial response (PR), stable disease (SD) or progressive disease (PD). The objective response rate (ORR) was defined as the proportion of patients who had a CR or PR that was confirmed with a subsequent scan at least four weeks following treatment. The disease control rate (DCR) reported the proportion of patients with tumor shrinkage or stabilization for at least four weeks, including cases with CR, PR and SD. Progression-free survival (PFS) was calculated from the date of the HIFU treatment until progression, death or the last follow-up appointment. Overall survival (OS) was calculated from the date of HIFU treatment until death or the last follow-up appointment. Complications such as pain, skin reactions, bleeding, urogenital system and digestive system damage were also analyzed, which were based on the Society of Interventional Radiology (SIR) Classification System for Complications by Outcome (27). August 2021 was used as the end date of the study for the PFS and OS data censorship.

### Statistics

All the data was presented using the mean ± standard deviation unless otherwise specified. The statistical analysis was performed using SPSS version 25.0. The actuarial survival was computed using the Kaplan-Meier method, prognostic factors were compared using the log-rank test and the Cox hazard proportion model was used in the multivariate analysis. The threshold for statistical significance was set at a *P*-value of equal to or less than 0.05.

## Results

### Clinical characteristics

A total of 217 cervical cancer patients received HIFU treatment at the Guizhou Provincial People’s Hospital from January 2010 to January 2021, of which 153 were included in this study in accordance with the aforementioned inclusion and exclusion criteria. Of the 153 patients, 52 cases represent patients with residual tumors that remained from the initial therapy and 101 patients experienced regional (from within the previous radiotherapy area) disease recurrence following irradiation (**Figure S1**). The clinicopathological data for these patients is presented in **Table 1**. All patients presented with stage IIA-IVA disease (predominantly squamous cell carcinoma) at initial diagnosis with an average patient age of 50.84 ± 10.99 years. The average lesion size was 1.80 ± 0.43 cm before HIFU treatment, with the uterine cervical or vaginal region being the most common site of the residual or recurrent lesions.

**Table 1:** Clinical characteristics for the cervical cancer patients prior to HIFU treatment.

Characteristics	Total (N = 153)	Residual tumor Before HIFU (N = 52)	Recurrence Before HIFU (N = 101)
Age (years), mean ± SD	50.84 ± 10.99	53.17 ± 10.13	49.63 ± 11.26
2009 FIGO stage at initial diagnosis, *N (%)*			
IIA	20 (13.1)	5 (9.6)	15 (14.9)
IIA1	4 (2.6)	0	4 (4)
IIA2	16 (10.5)	5(9.6)	11 (10.9)
IIB	14 (9.2)	3 (5.8)	11 (10.9)
III	74 (48.4)	27 (51.9)	47 (46.6)
IIIA	35 (22.9)	13 (25.0)	22 (21.8)
IIIB	39 (25.5)	14 (26.9)	25 (24.8)
IVA	45 (29.4)	17 (32.7)	28 (27.7)
Tumor Type, *N (%)*			
Squamous cell carcinoma	146 (95.4)	50 (96.2)	96 (95.0)
Adenosquamous carcinoma	7 (4.6)	2 (3.8)	5 (5.0)
ECOG performance status, *N (%)*			
0-1	145 (94.8)	49 (94.2)	96 (95.0)
2	8 (5.2)	3 (5.2)	5 (5.0)
Lesion size (cm), mean ± SD	1.80 ± 0.43	1.79 ± 0.40	1.81 ± 0.45
Lesion location, *N (%)*			
Cervix bed alone	81(52.9)	32 (61.5)	49 (48.5)
Pelvic lymph node	66 (43.1)	19 (36.5)	47 (46.5)
Cervix bed & Pelvic lymph node	6 (3.9)	1 (2.0)	5 (5.0)
Primary treatment, *N (%)*			
Definitive RT or CRT	105 (68.6)	52 (100.0)	53 (52.5)
RT equivalent dose to 2Gy			
<85Gy	15 (14.3)	7 (13.5)	8 (15.1)
≥85Gy	90 (85.7)	45 (86.5)	45 (84.9)
CT			
With CT	71 (67.6)	37 (71.2)	34 (64.2)
Without CT	34 (32.4)	15 (28.8)	19 (35.8)
Surgery followed by CRT	48 (31.4)	0	48 (47.5)
RT equivalent dose to 2Gy			
<85Gy	8 (16.7)	0	8 (16.7)
≥85Gy	40 (83.3)	0	40 (83.3)
CT			
With CT	38 (79.2)	0	38 (79.2)
Without CT	10 (20.8)	0	10 (20.8)

Abbreviations: Number (N), Standard Deviation (SD), International Federation of Gynecology and Obstetrics (FIGO), Eastern Cooperative Oncology Group (ECOG), gray (Gy), radiotherapy (RT), chemoradiotherapy (CRT) and chemotherapy (CT).

### Evaluation of HIFU treatment

**Figure 1:**
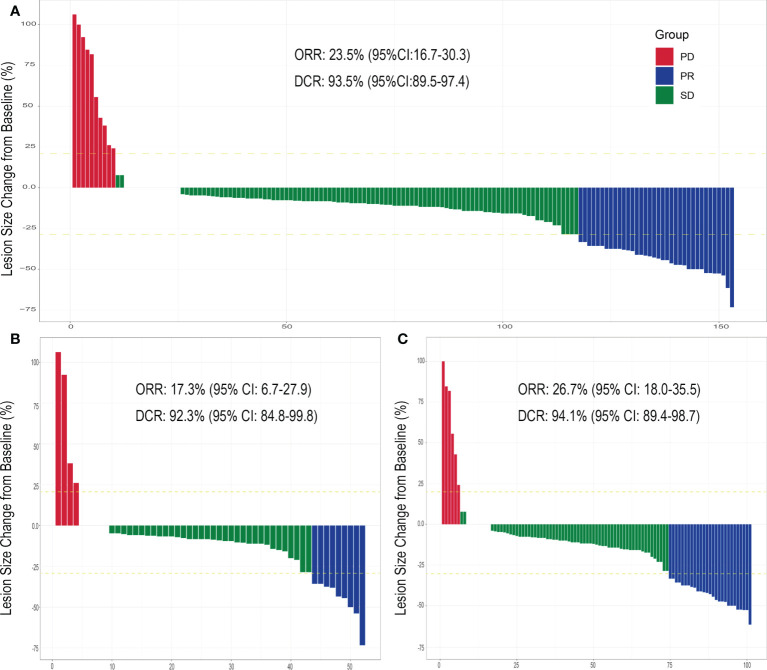
Evaluation of cervical tumor response after HIFU treatment. The index tumor size change from the baseline following HIFU treatment for **(A)** cervical cancer patients previously treated with CRT and experiencing residual tumors or recurrence (total) within a previously irradiated field, **(B)** patients experiencing residual tumors and **(C)** patients experiencing tumor recurrence. Abbreviations: partial response (PR), stable disease (SD), progressive disease (PD), objective response rate (OR), disease control rate (DCR) and confidence interval (CI).

**Figure 2:**
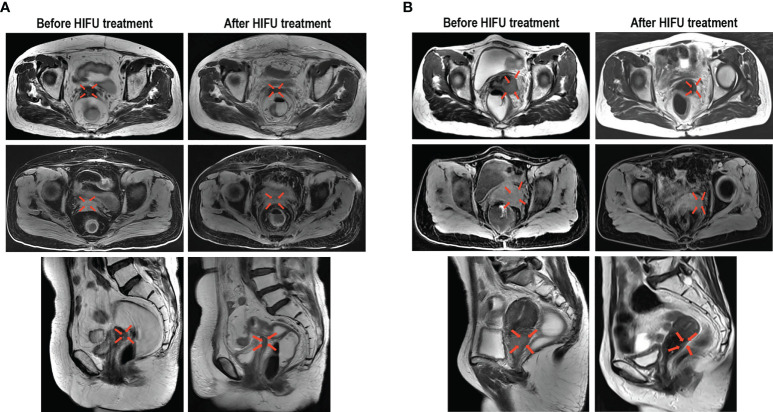
Representative images demonstrating the reduction of cervical cancer lesions following HIFU treatment. **(A)** Images from a 54-year-old patient with stage IIB cervical cancer before and after HIFU treatment, which display residual lesions that were previously treated with CRT. **(B)** Images from a 55-year-old patient with stage IIIB cervical cancer before and after HIFU treatment, which display recurrent lesions that occurred within an area that was previously irradiated. The transverse T2 MR images, transverse T1-fs MR images and sagittal T2 MR images all show that the tumor was significantly reduced.

Each patient received on average 9.64 ± 0.86 (range: 5-10) fractions within one HIFU ablation course. The average treatment power, treatment time and sonication time was shown in **Table 2**. None of the 153 patients experienced CR following the HIFU treatment, but PR in was observed for 36 cases (23.5%) and SD for 107 cases (69.9%). The ORR and DCR were 23.5% (95% Confidence Interval [CI], 16.7-30.3) and 93.5% (95% CI: 89.5-97.4), respectively. The changes in the index tumor size from the baseline are shown in **Figure 1**. Representative images displaying lesions before and after HIFU treatment were shown in **Figure 2**. Age, stage, initial treatment, biologically effective dose of radiotherapy, lesion location and size did not affect the disease control for these patients (**Figure S2**).

**Table 2:** HIFU ablation parameters and treatment response.

Parameters and Response	Total (N = 153)	Residual tumor Before HIFU(N = 52)	Recurrence Before HIFU(N = 101)
Number of fractions within one course, N± SD (range)	9.64 ± 0.86 (5-10)	9.60 ± 0.99 (6-10)	9.68 ± 0.81 (5-10)
Treatment duration (minutes), mean ± SD (range)	26.53 ± 1.40 (24-29)	26.59 ± 1.41 (24-29)	26.56 ± 1.40 (24-29)
Sonication time (seconds), mean ± SD (range)	471.33 ± 44.52 (420-540)	469.50 ± 43.52 (420-540)	472.02 ± 44.22 (420-540)
Treatment intensity (seconds/hours), mean ± SD (range)	1068.01 ± 111.35 (868.97-1350)	1062.18 ± 110.77 (868.97-1350)	1068.95 ± 111.85 (868.97-1350)
Average power (watts), mean ± SD (range)	647.45 ± 70.99 (500-800)	672.05 ± 72.72 (500-800)	674.16 ± 70.16 (500-800)
HIFU Therapy Outcome, *N (%)*			
CR	0	0	0
PR	36 (23.5)	9 (17.3)	27 (26.7)
SD	107 (69.9)	39 (75.0)	68 (67.3)
PD	10 (6.5)	4 (7.7)	6 (5.9)

Abbreviations: Number (N), Standard Deviation (SD), complete response (CR), partial response (PR), stable disease (SD) and progressive disease (PD).

### Survival analysis

During this study the mPFS was 17.0 months (95% CI: 15.5-18.5) and the mOS was 24.5 months (95% CI: 22.3-26.7). The PFS rate was 72.5% for 12 months and 14.4% for 24 months. The OS rate was 46.4% and 7.2% for 24 and 48 months, respectively. The loss to follow-up rate was 8.5% and the median time to follow-up was 36.0 months (95% CI: 34.9-37.1).

**Figure 3:**
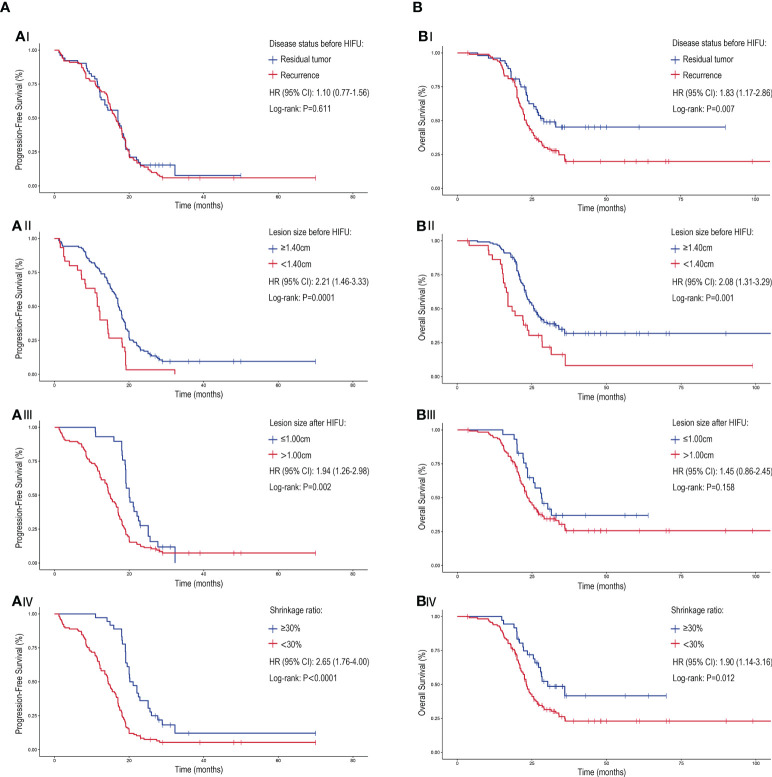
The PFS and OS associated with of different disease patterns and lesion characteristics for cervical patients treated with HIFU. The **(A)** PFS and **(B)** OS of cervical cancer patients treated with HIFU. (A/BI) The response to HIFU for previously irradiated patients that experienced residual disease compared to recurrence. Patient outcome when stratified for (A/BII) lesion size prior to HIFU treatment, (A/BIII) lesion size after HIFU treatment and (A/BIV) lesion shrinkage ratio (comparing the lesion size change before and after HIFU treatment). Crosses were used to denote censored patients. Hazard ratio (HR), confidence interval (CI).

The survival analysis demonstrated that the mPFS for patients with residual or recurrent lesions ≥1.40 cm was significantly higher than those with lesions less than 1.40 cm (17.2 months [95%CI: 15.5-18.5] versus 11.5 months [95%CI: 10.6-12.4]; *P*=<0.0001). After treatment, patients with a lesion size of ≤1.00cm had a higher mPFS than patients with >1.00cm (20.0 months [95% CI: 18.2-21.6] versus 14.9 months [95% CI: 13.4-16.4]; *P*=0.002) and patients with a shrinkage rate of ≥30% had a higher mPFS than those with <30% (20.2 months [95% CI: 17.7-22.7] vs. 14.4 months [95% CI: 12.9-15.9]; *P*=<0.0001) (**Figure 3A**).

**Figure 4:**
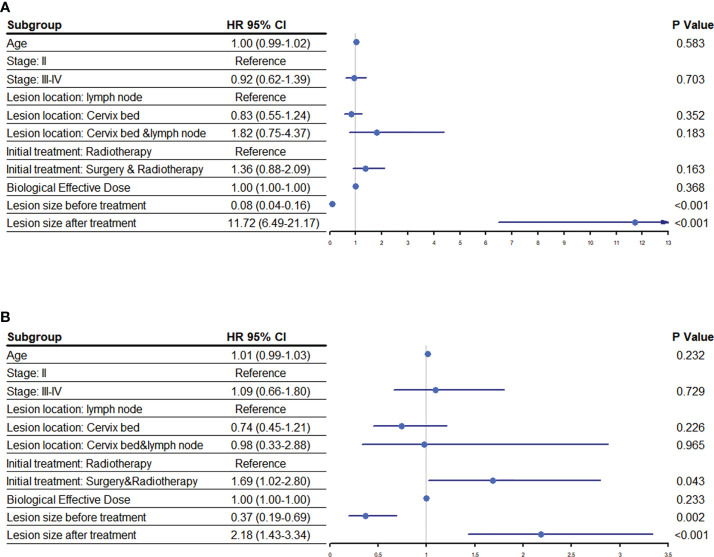
Risk factors associated with prognosis following HIFU. An analysis to determine the association of various risk factors with the **(A)** progression free survival and **(B)** overall survival of cervical cancer patients following HIFU treatment. Abbreviations: hazard ratio (HR), confidence interval (CI).

The mOS for patients with residual tumor prior to HIFU was higher than those with disease recurrence (29.0 months vs. 23.1 months [95% CI: 21.4-24.8]; *P*=0.007); but the mPFS of patients with the two disease patterns were similar (*P*=>0.05). Correspondingly, patients with lesions ≥1.40 cm before treatment and a lesions shrinkage ratio of ≥30% after treatment had better mOS. No statistical difference in the mOS for patients with different lesion sizes following the treatment was observed (*P*=>0.05) (**Figure 3B**). A multivariate analysis showed that the lesion size before or after HIFU treatment was significantly related to PFS and OS. Moreover, patients whose initial treatment was radiotherapy or CRT after surgery had worse survival when compared to those who received radical radiotherapy or CRT (Hazard ratio: 1.69 [95% CI: 1.02-2.80]; *P*=0.043) (**Figure 4**).

### Safety

The treatment-related adverse events for all the patients were considered to be minor complications. Of the 153 patients, 19 patients (12.4%) had grade A/B skin burns, 18 patients (11.8%) had grade A abdominal pain and 9 patients (5.9%) had grade A vaginal discharge. It is noteworthy that all the patients with vaginal bleeding had this symptom prior to the HIFU treatment, and that the bleeding did not worsen following treatment. No adverse events that were grade C or higher occurred in any of the patients. Additionally, there were no reports of vesicovaginal fistula, rectovaginal fistula, ileus, hemorrhage, infection or damage to other organs (**Table S1**). The occurrence of complications was not affected by age, stage, initial treatment, biologically effective dose of radiotherapy or lesion location and size (**Figure S3**).

## Discussion

While prophylactic HPV vaccination programs have led to a significant reduction in cervical cancer incidence and mortality in developed countries, the disease burden of cervical cancer in underdeveloped countries remains high and the treatment of advanced disease is problematic. The publication of the KEYNOTE-826 study indicated that pembrolizumab combined with chemotherapy ± bevacizumab is the preferred treatment option for patients with PD-L1-positive persistent, recurrent or metastatic cervical cancer. However, the mPFS was only 10.4 months for these patients, even with the addition of immunotherapy (28). More efficient and personalized treatment options are still required. Previous studies have demonstrated the feasibility of HIFU for the treatment of cervical cancer, but the reports are sporadic and often do not contain information regarding adverse effects or survival data (29). It was therefore important to assess these aspects of HIFU application for patients with residual or recurrent cervical cancer following CRT, which has been detailed in the study contained herein.

The ORR and DCR after HIFU treatment were 26.1% and 93.5%, respectively. The mPFS and mOS of patients reached 17.0 months and 24.5 months, respectively. Moreover, treatment-related complications were few and mild, and patient recovery following treatment was generally fast. Even patients with an advanced age or poor physical condition were able to tolerate the HIFU therapy (the oldest patient in this study was 84 years old). This is due to the relative safety of the HIFU treatment, which leaves tissue immediately outside the target area mostly intact (30, 31). Indeed, we have observed that an outpatient HIFU service can be offered for patients who are relatively asymptomatic at the time of consultation and have no obvious adverse reactions during the treatment. This is desirable because the outpatient service helps reduce the physical and economic burden for these patients. Furthermore, this therapy can be conducted regardless of the patient PD-L1 expression status. Our study shows that local HIFU therapy may be a good option for patients with residual or recurrent tumors located in the pelvis.

Previous studies have shown that re-irradiation for cervical cancer patients may cause surrounding healthy tissues to be exposed to an intolerable dose that can result in serious complications, such as radiation enteritis, intestinal perforation and rectovaginal fistula. Whereas, the study herein demonstrated that the survival and complication rate after HIFU treatment was not related to the previous exposure dose of patient. This suggests that the application of HIFU treatment is not limited to the level of the previous radiation dose and the tolerance of the surrounding healthy tissues. Nevertheless, brachytherapy may be an option for carefully selected patients with smaller central lesions (<2 cm) (32–35). The use of image-guided radioactive ^125^I seed has been reported as a method for the treatment of patients with recurrent disease after external beam radiotherapy, the local control rate was 88.9%, and the median local PFS and mOS were 7.5 months and 11.5 months, respectively (36). Different methods of external beam radiotherapy have also been proposed for these patients, such as stereotactic body radiotherapy and proton therapy, however the sample sizes included in these studies have been small so far and thus further demonstration of efficacy and safety is needed (37).

The prognosis for patients with tumor recurrence has generally been considered to be worse than those with residual disease, which is consistent with the observations herein, where the survival time was shorter for patients experiencing recurrence. However, the mPFS of the two categories was similar, which suggests that HIFU treatment has the same local control capability for lesions resulting from residual and recurrent lesions. Additionally, patients with smaller lesions after HIFU had a better mPFS, which is consistent with previous reports (18, 38, 39). Moreover, patients with greater lesion shrinkage after HIFU treatment have a higher mPFS and mOS, which supports the notion that HIFU treatment was efficacious for cervical cancer patients with residual disease or recurrence following CRT.

It is interesting to note that previous studies have suggested that patients with larger residual or recurrent tumors had a poorer prognosis, regardless of whether they were treated with surgery, radioactive seed implantation or brachytherapy (11, 33, 40). Whereas the patient’s described herein with lesions ≥1.4cm had a significantly greater mPFS and mOS than those with lesions <1.4cm. This may be related to the greater ability to obtain an accurate target location for large lesions and/or the higher target dose used. However, this could be due to an insufficient sample size and/or insufficient follow-up time and therefore a rigorous large-sample prospective study would be required to validate this observation.

This retrospective analysis was a single-center study, which does introduce the possibility that there were confounding factors that were not considered during the analysis. Nevertheless, this study demonstrates that HIFU treatment could provide a complementary method for the treatment of cervical cancer patients who experience a poor outcome following CRT. It is interesting that the multivariate analysis showed that patients whose initial treatment was surgery followed by radiotherapy or CRT had a higher risk of death than those who received radical radiotherapy or CRT. This notion requires further confirmation and mechanistic exploration. It also further highlights the need for a larger prospective multi-center randomized trial to verify these results and more precisely define the patient subgroups that would benefit the most from HIFU treatment.

## Conclusions

Our research shows that HIFU treatment can significantly reduce the size of lesions in cervical cancer patients with residual disease or recurrence following CRT and is capable of increasing the local control rate and survival time. The non-invasive nature of HIFU means that the treatment-related side effects are less frequent, which enables the provision of an efficacious highly tolerable therapy to a group of patients who are often not able to receive other conventional therapies.

## Data availability statement

The datasets used and/or analyzed during the current study are available from the corresponding author upon reasonable request.

## Ethics statement

Approval of the treatment was obtained from the Guizhou Province People's Hospital Institutional Review Board Committee. Each participant provided their written informed consent.

## Author contributions

QZ, FT, and TN: Investigation, Formal analysis, Data Curation, Writing-Original Draft. YC and YL: Resources. JW: Visualization. WZ, ZF, and XL: Software, Validation. ST: Project administration. YZ: Conceptualization, Methodology, Writing-Review and Editing, Supervision. All authors reviewed and approved the final manuscript.

## Funding

This project was supported by Higher-level New-type Talent Foundation of Guizhou Province [GZSYQCC (2016) 003], Health Commission Science and Technology Foundation of Guizhou Province (gzwjkj 2020-1-032; gzwkj 2022-028) and Youth Fund of Guizhou Provincial People's Hospital (GZSYQN [2021] 03).

## Acknowledgments

We thank all the patients and family members for participating in this study. We also thank their other clinical practitioners and nurses for providing invaluable support. This study was presented for online publication at the 2022 American Society of Clinical Oncology (ASCO) Annual Meeting on May 26, 2022 (ASCO.org/abstracts).

## Conflict of interest

The authors declare that the research was conducted in the absence of any commercial or financial relationships that could be construed as a potential conflict of interest.

## Publisher’s note

All claims expressed in this article are solely those of the authors and do not necessarily represent those of their affiliated organizations, or those of the publisher, the editors and the reviewers. Any product that may be evaluated in this article, or claim that may be made by its manufacturer, is not guaranteed or endorsed by the publisher.
