# Supplementary material for: Salvage high intensity focused ultrasound for residual or recurrent cervical cancer after definitive chemoradiotherapy
Source: Front Immunol. 2022 Oct 17;13:995930. doi: 10.3389/fimmu.2022.995930 (PMC9618866; doi:10.3389/fimmu.2022.995930)
Supplement: Supplementary file 1 [file DataSheet_1.docx]

**Figure S1. Flowchart of the study design.**

**
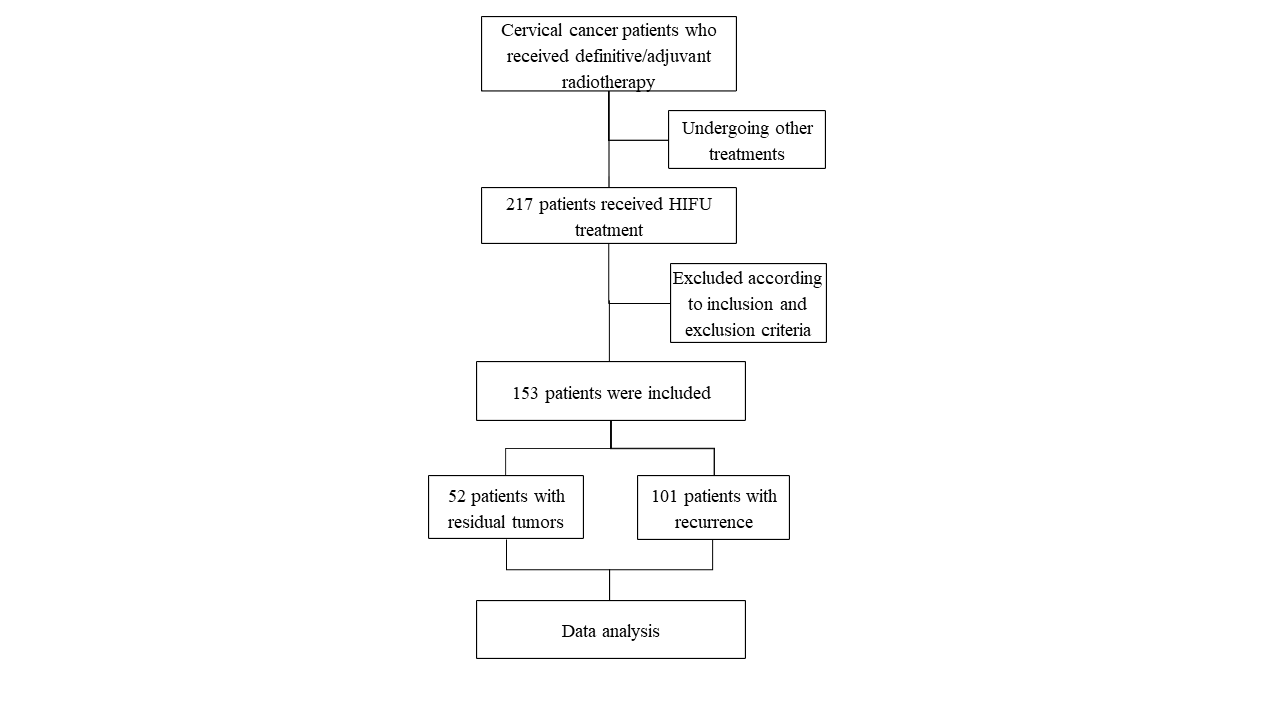
**

**Figure S2. Risk factors associated with disease control following HIFU.**

**
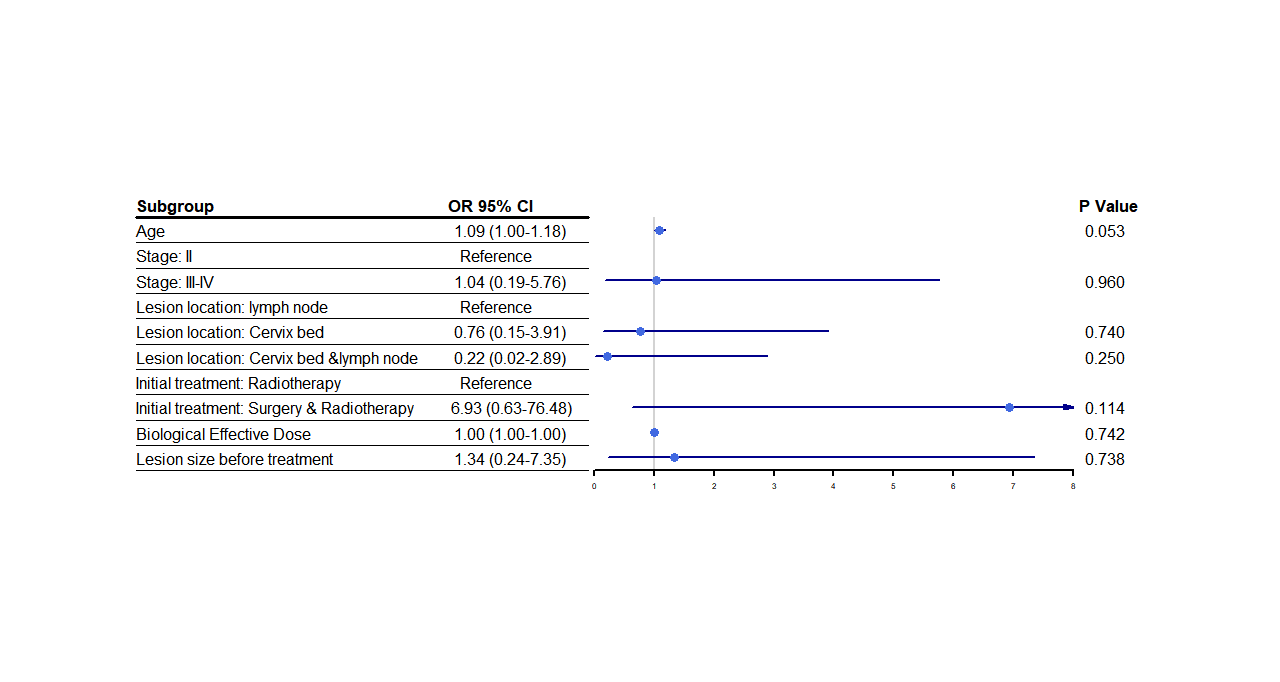
**

An analysis to determine the association of various risk factors with the disease control (complete response, partial response and stable disease) of cervical cancer patients following HIFU treatment. Abbreviations: odds ratio (OR), confidence interval (CI).

**Figure S3. Risk factors associated with the occurrence of complications following HIFU.**


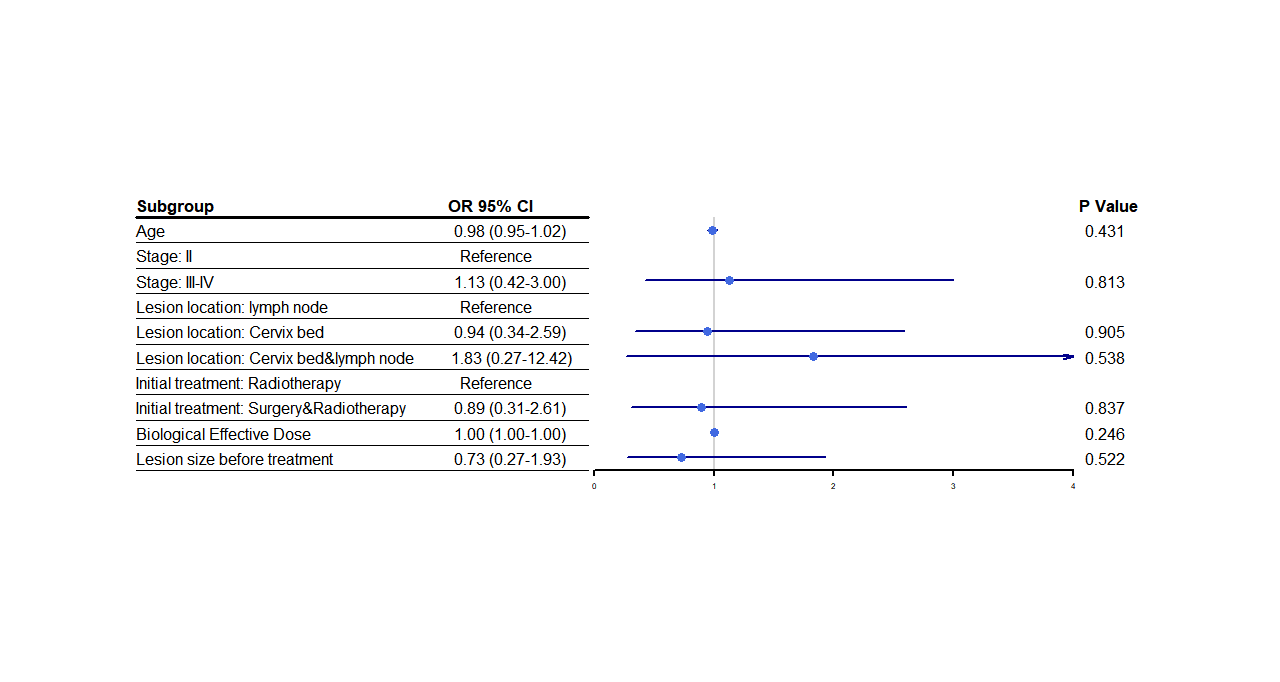


An analysis to determine the association of various risk factors with the occurrence of complications in cervical cancer patients following HIFU treatment. Abbreviations: odds ratio (OR), confidence interval (CI).

**Table S1. Adverse events experienced by cervical cancer patients after HIFU treatment.**

| **Adverse Event** | **Total**  **N/153 (%)** | | | **Residual tumor Before HIFU**  **N/52 (%)** | | | **Recurrence Before HIFU**  **N/101 (%)** | | |
| --- | --- | --- | --- | --- | --- | --- | --- | --- | --- |
|  | Class A | Class B | A&B | Class A | Class B | A&B | Class A | Class B | A&B |
| Skin burns | 10 (6.5) | 9 (5.9) | 19 (12.4) | 7 (13.5) | 5 (9.6） | 12 (23.1) | 3 (3.0) | 4 (4.0) | 7 (6.9) |
| Abdominal pain | 18 (11.8) | 0 | 18 (11.8) | 10 (19.2) | 0 | 10 (19.2) | 8 (7.9) | 0 | 8 (7.9) |
| Vaginal discharge | 9 (5.9) | 0 | 9 (5.9) | 4 (7.7） | 0 | 4 (7.7) | 5 (5.0) | 0 | 5 (5.0) |
| More severe vaginal bleeding | 0 | 0 | 0 | 0 | 0 | 0 | 0 | 0 | 0 |
| Abdominal distension | 0 | 0 | 0 | 0 | 0 | 0 | 0 | 0 | 0 |
| Sacrococcygeal pain | 0 | 0 | 0 | 0 | 0 | 0 | 0 | 0 | 0 |
| Infection | 0 | 0 | 0 | 0 | 0 | 0 | 0 | 0 | 0 |
| Fistulas | 0 | 0 | 0 | 0 | 0 | 0 | 0 | 0 | 0 |
| Hematochezia | 0 | 0 | 0 | 0 | 0 | 0 | 0 | 0 | 0 |
| Ileus | 0 | 0 | 0 | 0 | 0 | 0 | 0 | 0 | 0 |
| Ureteral injury | 0 | 0 | 0 | 0 | 0 | 0 | 0 | 0 | 0 |
| Urinary tract infection | 0 | 0 | 0 | 0 | 0 | 0 | 0 | 0 | 0 |
| Urinary incontinence | 0 | 0 | 0 | 0 | 0 | 0 | 0 | 0 | 0 |

Adverse Event grades are based on the Society of Interventional Radiology (SIR) Classification System for Complications by Outcome: Class A minor complication) = no therapy, no consequence and Class B (minor complication) = nominal therapy, no consequence. No events with grades higher than B occurred (class C-F represent major complication). Abbreviation: Number (N).
